# Infliximab response associates with radiologic findings in bio-naïve Crohn’s disease

**DOI:** 10.1007/s00330-023-09542-y

**Published:** 2023-03-16

**Authors:** Chen Yueying, Feng Jing, Feng Qi, Shen Jun

**Affiliations:** 1 State Key Laboratory for Oncogenes and Related Genes, Key Laboratory of Gastroenterology & Hepatology, Ministry of Health, Division of Gastroenterology and Hepatology, Ren Ji Hospital, School of Medicine, Shanghai Jiao Tong University, Shanghai Cancer Institute, Shanghai Institute of Digestive Disease, Shanghai, China; 2 Department of Gastroenterology and Hepatology, Zhongshan Hospital, Fudan University, Shanghai, China; 3 Department of Radiology, Renji Hospital, School of Medicine, Shanghai Jiao Tong University, 160 Pu Jian Road, Shanghai, China

**Keywords:** Crohn’s disease, Radiomics, Magnetic resonance enterography, Infliximab

## Abstract

**Objectives:**

Since a reliable model for predicting infliximab (IFX) benefits in bio-naïve Crohn’s disease (CD) is still lacking, we constructed a magnetic resonance enterography (MRE)-based model to predict the risk of loss of response to IFX in bio-naïve patients with CD.

**Methods:**

This retrospective multicenter study enrolled 188 bio-naïve patients with CD who underwent MRE before IFX therapy. Therapeutic outcomes were determined based on clinical symptoms and endoscopic findings within 52 weeks. The areas of bowel wall segmentation were decided by two experienced radiologists in consensus. Texture features were extracted using the least absolute shrinkage and selection operator, and a radiomic model was built using multivariate logistic regression. The model performance was validated by receiver operating characteristic, calibration curve, and decision curve analysis.

**Results:**

The area under the curve of radiomic model was 0.88 (95% confidence interval: 0.82–0.95), and the model provided clinical net benefit in identifying the loss of response to IFX and exhibited remarkable robustness among centers, scanners, and disease characteristics. The high-risk patients defined by the radiomic model were more likely to develop IFX nonresponse than low-risk patients (all *p* < 0.05).

**Conclusions:**

This novel pretreatment MRE-based model could act as an effective tool for the early estimation of loss of response to IFX in bio-naïve patients with CD.

**Key Points:**

*• Magnetic resonance enterography model guides infliximab therapy in Crohn’s disease.*

*• The model presented significant discrimination and provided net clinical benefit.*

*• Model divided patients into low- and high-risk groups for infliximab failure.*

**Supplementary Information:**

The online version contains supplementary material available at 10.1007/s00330-023-09542-y.

## Introduction

Crohn’s disease (CD) is a chronic and recurrent inflammatory bowel disease, and its prognosis has improved in recent years with biologics as the milestone for therapy. Infliximab (IFX) is the first anti-tumor necrosis factor (TNF) α agent. However, 13–40% and 23–46% of patients with CD exhibit primary loss of response (PLR) and secondary loss of response (SLR) to IFX, respectively [1, 2]. The outcome of IFX therapy is mainly assessed by periodic enteroscopy, while some patients cannot receive timely therapies after IFX failure due to the limitations of enteroscopy. Therefore, it is vital to predict the risk of nonresponse prior to IFX treatment. In addition to clinical characteristics [3], other biomarkers, including bile acid metabolites [4], genetic markers [5], and gut microbiota [6], have been identified in IFX response prediction, most of which were obtained by invasive examinations. Magnetic resonance enterography (MRE) is a valuable adjunct for assessing luminal and mural manifestations in the initial stages of CD [7, 8]. The MRE characteristics are highly predictive of the requirement for surgery in stricturing CD [9], and patients with MRE inflammation are more vulnerable to treatment modification [10]. Previous studies have revealed that creeping fat and location of CD are relevant predictors of the effectiveness of anti-TNF therapy [11], and the reduced apparent diffusion coefficient (ADC) values were related to anti-TNF treatment failure in stricturing CD [12]. These findings demonstrated the promising role of MRE in predicting the efficacy of IFX treatment for CD. As a machine learning tool, texture analysis (TA) of magnetic resonance imaging (MRI) has been applied to evaluate disease staging [13], and predict rectal cancer recurrence after chemoradiotherapy [14]. Additionally, the texture features based on MRE can detect fibrosis and strictures in CD [15]. However, the role of MRE-based TA in assessing the outcome of anti-TNF therapy in CD remains elusive. Our study aimed to construct an MRE-based model and identify the predictive role of texture and clinical features in estimating nonresponse to IFX in CD.

## Materials and methods

### Patients and study design

We enrolled 188 patients diagnosed with CD according to the European Crohn’s and Colitis Organization (ECCO) guidelines in Renji Hospital from the east and west campuses, between January 2013 and August 2020 [16]. 112 and 76 patients were randomly allocated to the training and validation cohorts. Ethical approval was obtained from the Institutional Review Board of Shanghai Jiaotong University School of Medicine, Renji Hospital Ethics Committee (KY2020-115). Bio-naïve patients who underwent MRE before IFX administration were included, whereas patients with anti-TNF therapy exposure history, unavailability of 5 mg/kg IFX therapy, or inadequate MRE data were excluded from the study.

### Definition of clinical endpoint

The outcomes of IFX therapy were assessed by an experienced multidisciplinary team based on clinical symptoms and endoscopic findings. Clinical response to IFX therapy was considered as a Harvey-Bradshaw Index (HBI) reduction of > 2 points from baseline [17]. The failure of IFX was divided into PLR and SLR. PLR was defined as a lack of clinical response (decrease in HBI ≥ 3 points) at week 14, whereas SLR was defined as a relapse after an initial response to IFX within 52 weeks requiring alternative treatments (other biological agents, corticosteroids, immunosuppressants, dose escalation, and intestinal resection), HBI > 5 or an increase in the HBI ≥ 3 points, and/or mucosal inflammation (a reduction from baseline in simple endoscopic score for CD (SES-CD) < 50% or SES-CD ≥ 3).

### Procedure of magnetic resonance enterography (MRE)

Patients received bowel preparation 8 h before their MRE examination. All patients were instructed to drink 1000–1200 mL of polyethylene glycol (Wanghe Pharma) 45 min before scanning, and 10 mg of anisodamine (First Biochemical Pharma) was injected through the gluteus maximus 10 min before examination. MRE examinations were performed using a 1.5-T GE scanner (Optima MR360; GE HealthCare) or a 3-T Philips scanner (Ingenia, Philips). All images were obtained using routine MRE protocol in supine position. The parameters of MR sequences are presented in Supplementary Tables 1. In contrast-enhanced phase, 15 mL of Magnevist (Bayer Schering) was injected through dorsal palmar vein. For image analysis, diffusion-weighted imaging (DWI), enteric-phase T1-weighted image with fat suppression (55–70 s after contrast administration) (T1WI), T2-weighted image (T2WI), and T2-weighted image with fat suppression (T2WI-FS) were selected.

### Volume-of-interest (VOI) segmentation and feature extraction

**Fig. 1:**
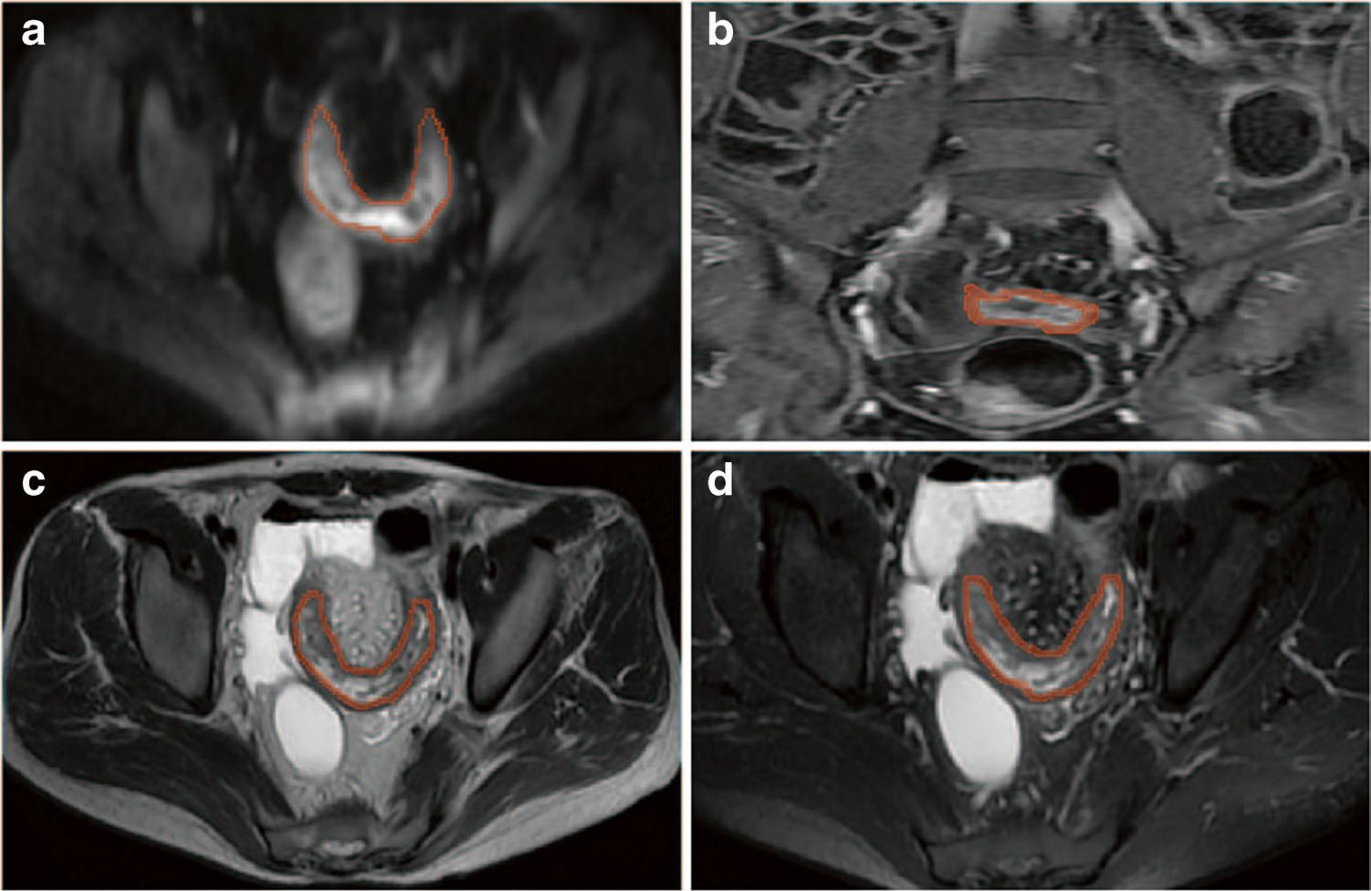
Examples of volume-of-interest regions drawn on MRE images of different sequences (red semi-transparent area). **a** diffusion-weighted image. **b** contrast-enhanced T1-weighted image. **c** T2-weighted image. **d** T2-wesighted image with fat suppression

All VOIs were segmented blindly by two radiologists with over 13 years of experience. To verify the reliability of radiomic features, only those with an intraclass correlation coefficient (ICC) > 0.8 were selected as candidate predictors. VOIs were drawn in the same intestinal segments with the most severe inflammation on four sequences (contrast-enhanced T1WIs, T2WIs, T2WI-FS, and DWI) of each patient prior to IFX therapy (Fig. 1). The criteria for determining active inflammation were as follows: bowel wall thickening with edema on T2WI-FS imaging; bowel wall with ulcers on mucosal surface; marked enhancement of bowel wall; bowel wall with penetrating disease or active adjacent mesenteric inflammation. Features were extracted using PyRadiomics, and VOIs were resampled into 3 × 3 × 3 mm^3^, and the gray levels were distributed into 25 levels. A total of 412 radiomic features were extracted from T1WI, T2WI, T2WI-FS, and DWI of each patient. All radiomic variables were normalized by *z*-score transformation.

### Feature selection

The correlations among radiomic features with ICC > 0.8 were analyzed by Spearman’s test, and those with a correlation coefficient of > 0.8 were eliminated. Eventually, 111 features were obtained, comprising shape [23], first-order [15], and textural features (73) (Supplementary Table 2). Features with nonzero coefficients in least absolute shrinkage and selection operator (LASSO) were incorporated into the models. The clinical characteristics were screened by univariable logistic regression.

### Feature definitions

GLSZM is defined as the groups of connected voxels sharing the same gray level intensity by quantifying the distance between similar zones, and the less homogeneous tissue hass shorter distances and fewer similar gray levels [18]. Skewness is a first-order feature that indicates the asymmetry and brightness of the histogram, and the absolute value indicates the degree of deviation [19]. The increased value of T2W_SDLGLE indicates a more heterogeneous and lower gray level texture [20].

### Model development and validation

Multivariate logistic regression analysis with the forward stepwise algorithm was applied in model development, and the model fit was assessed using the minimal Akaike information criterion (AIC). Receiver operating characteristic (ROC) curve was used to evaluate the model discrimination, and the area under the curves (AUCs) were compared using DeLong’s test. The accuracy and efficiency of the model were estimated using a calibration curve and decision curve analysis (DCA). Integrated discrimination improvement (IDI) was used to measure the quantitative improvement of the model introduced by the addition of variables.

### Outcomes of IFX therapy in low- and high-risk groups based on radiomic model

The radiomic scores based on the model were dichotomized into a cutoff value, and the optimal value was chosen using the maximally selected rank statistics method. Then, patients were allocated into low- and high-risk groups for IFX nonresponse. Kaplan–Meier survival analysis and the log-rank test were performed to evaluate IFX efficacy within 52 weeks between the two groups.

### Statistical analysis

The sample size calculation criteria were as follows: 80% power, AUC = 0.80, two-sided significance level set at 0.05, with an allocation ratio of 1. Student’s *t*-test and the Mann–Whitney *U* test were used to analyze normally and non-normally distributed continuous variables, respectively, and the values are presented as mean ± standard deviation or median with interquartile range. Categorical variables were compared using the chi-square test. Interobserver agreement between paired evaluations of MRE by two radiologists was performed through kappa statistics. Univariate logistic regression was applied to analyze the relationships between different factors and the outcome. Multivariable regression analysis was used to search the model that best fit the data. Odd ratios (OR) and 95% confidence intervals (CI) were described for models. Adjustment for multiple comparisons was carried out using the Benjamini–Hochberg (BH) procedure to control the false discovery rate. Statistical tests were performed using SPSS 24.0 and R statistical software (version 3.6.3), and a two-sided *p* < 0.05 was considered statistically significant.

## Results

### Clinical characteristics

**Fig. 2:**
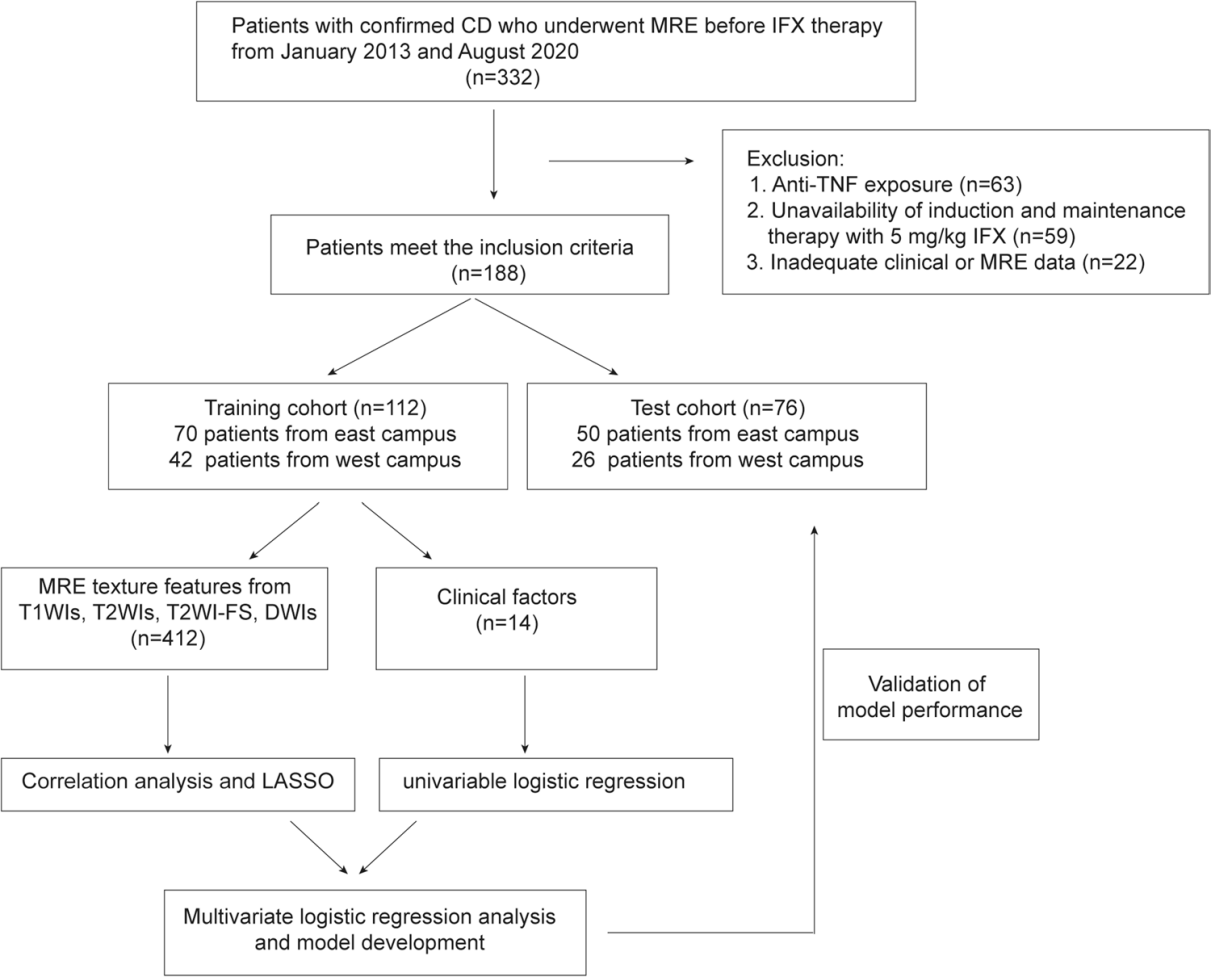
Flowchart of the study population and design

A flowchart of the study is depicted in Fig. 2. Among the 188 enrolled patients who received IFX therapy, 43 (38.4%) and 21 (27.6%) nonresponders were in the training and validation cohorts, respectively. Table 1 presents the demographic and clinical characteristics between the two cohorts with all *p* values > 0.05, which revealed that the patients in the two cohorts had a balanced distribution of baseline characteristics.

**Table 1:** Demographic and clinical characteristics of patients with Crohn’s disease in the training cohort and validation cohort

Characteristics	Train (*n* = 112)	Test (*n* = 76)	*p* value
Sex (male/female, *n*)	80/32	60/16	0.25
Age, years (mean ± SD)	31.33 ± 11.08	32.01 ± 10.98	0.99
Duration of the disease, months, median (IQR)	12 (5.25–48)	24 (5.25–60)	0.44
BMI, kg/m^2^ (mean ± SD)	19.98 ± 3.06	20.68 ± 3.41	0.43
Prior surgery, *n* (%)	31 (27.68%)	18 (23.68%)	0.54
Smoking at first dose of IFX, *n* (%)	6 (5.36%)	3 (3.95%)	0.66
Location of specimen, *n* (%)			0.96
Small bowel	52 (46.43%)	35 (46.05%)	
Colon	60 (53.57%)	41 (53.95%)	
Phenotype, *n* (%)			0.35
Inflammatory	57 (50.89%)	44 (39.29%)	
Stricturing and/or penetrating	55 (49.11%)	32 (60.71%)	
Perianal fistulas, *n* (%)	74 (66.07%)	49 (64.47%)	0.82
CRP, mg/L, median (IQR)	2.41 (0.51–15.9)	3.65 (0.57–11.78)	0.51
ESR, mm/h, median (IQR)	12.5 (6–29)	13 (6.25–23)	0.99
Hb, g/L, median (IQR)	126.5 (110.5–137)	130.5 (114.25–141)	0.22
Alb, g/L (mean ± SD)	40.57 ± 6.31	42.25 ± 5.9	0.76
PLT, × 10^9^/L, median (IQR)	276.5 (215.5–336.75)	265.5 (213.5–329)	0.64
HBI, median (IQR)	6 (5–8)	6 (4–7)	0.13
SES-CD, median (IQR)	12 (7–17)	12 (7.25–15)	0.26

*IQR*, interquartile range; *SD*, standard deviation; *BMI*, body mass index; *CRP*, C-reactive protein; *ESR*, erythrocyte sedimentation rate; *Hb*, hemoglobin; *Alb*, albumin; *PLT*, platelets; *HBI*, Harvey-Bradshow Index; *SES-CD*, Simple Endoscopic Score for Crohn’s Disease

### Feature selection and model development

**Fig. 3:**
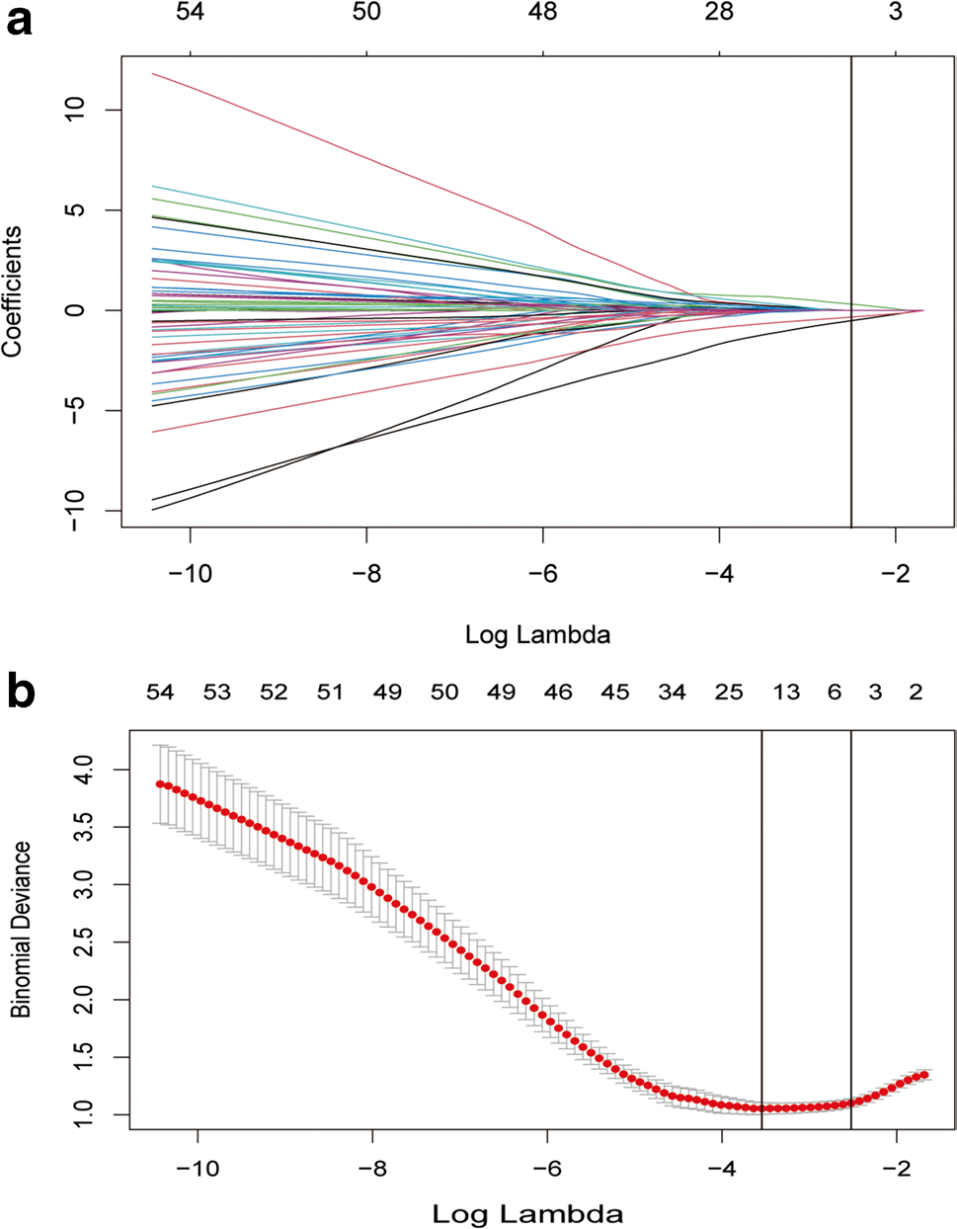
MRE features selection by the LASSO and tenfold CV. **a** Dotted vertical lines were drawn at the optimal *λ* of 0.08 with log (*λ*) =  − 2.526 based on minimum criteria. **b** LASSO coefficient profiles of the 111 radiomics features; three with nonzero coefficients were selected

The 111 radiomic features were reduced to three candidate features with the optimum *λ* = 0.08 identified by LASSO (Fig. 3), including DWI_GLSZM_SALGLE (reflects the small area low-gray-level emphasis of gray level small zone matrix in the DWIs), T1W _Skewness (indicates the asymmetry of the histogram in the T1WIs), and T2W_GLDM_SDLGLE (reflects the small dependence low-gray-level emphasis of the gray level difference matrix in the T2WIs). Body mass index (BMI) (*p* = 0.02) and disease phenotype (*p* = 0.02) were significant clinical variables in the univariate logistic regression (Supplementary Table 3). Therefore, we built a radiomic model (model 1) including only radiomic features, and a combined model (model 2) comprising radiomic and clinical features based on the minimal AIC (Tables 2 and 3). The formulas of two models are as follows: Predicted probability = 1 / (1 + e^−f(x)^).

**Table 2:** Multivariate regression analyses of the radiomic model and combined model. Radiomic model including DWI_GLSZM_SALGLE, T1_Firstorder_Skewness, and T2_GLDM_SDLGLE

Variables	*β*	OR (95% CI)	Se	*p*	Adj.*p*
DWI_GLSZM_SALGLE	− 2.06	0.13 (0.04–0.40)	0.58	0.000	0.000
T1_Firstorder_Skewness	− 1.05	0.35 (0.17–0.71)	0.36	0.004	0.004
T2_GLDM_SDLGLE	1.140	3.13 (1.53–6.40)	0.37	0.002	0.003

*OR*, odds ratio; *CI*, confidence interval; *Se*, standard error; *Adj.p*, adjust *p* value

**Table 3:** Multivariate regression analyses of the radiomic model and combined model. Combined model including BMI, disease behavior, DWI_GLSZM_SALGLE, T1_Firstorder_Skewness, and T2_GLDM_SDLGLE

Variables	*β*	OR (95% CI)	Se	*p*	Adj.*p*
BMI	− 0.12	0.89 (0.73–1.07)	0.10	0.216	0.27
Disease behavior	0.37	1.45 (0.51–4.16)	0.54	0.488	0.488
DWI_GLSZM_SALGLE	− 2.01	0.13 (0.04–0.42)	0.59	0.001	0.005
T1_Firstorder_Skewness	− 0.96	0.38 (0.19–0.79)	0.37	0.009	0.015
T2_GLDM_SDLGLE	1.10	3.02 (1.47–6.19)	0.37	0.003	0.008

*BMI*, body mass index; *OR*, odds ratio; *CI*, confidence interval; *Se*, standard error; *Adj.p*, adjust *p* value

$$\mathrm{f }(\mathrm{x})\mathrm{ in model }1\hspace{0.17em}=\hspace{0.17em}\hspace{0.17em}-\hspace{0.17em}1.25\hspace{0.17em}-\hspace{0.17em}2.06\hspace{0.17em}\times \hspace{0.17em}\mathrm{DWI}\_\mathrm{GLSZM}\_\mathrm{SALGLE}\hspace{0.17em}-\hspace{0.17em}1.05\hspace{0.17em}\times \hspace{0.17em}\mathrm{T}1\mathrm{W}\_\mathrm{Skewness}\hspace{0.17em}+\hspace{0.17em}1.14\hspace{0.17em}\times \hspace{0.17em}\mathrm{T}2\mathrm{W}\_\mathrm{GLDM}\_\mathrm{SDLGLE}$$

$$\mathrm{f }(\mathrm{x})\mathrm{ in model }2\hspace{0.17em}=\hspace{0.17em}0.99\hspace{0.17em}-\hspace{0.17em}0.12\hspace{0.17em}\times \hspace{0.17em}\mathrm{BMI}\hspace{0.17em}+\hspace{0.17em}0.37\hspace{0.17em}\times \hspace{0.17em}\mathrm{disease phenotype}\hspace{0.17em}-\hspace{0.17em}0.20\hspace{0.17em}\times \hspace{0.17em}\mathrm{DWI}\_\mathrm{GLSZM}\_\mathrm{SALGLE}\hspace{0.17em}-\hspace{0.17em}0.96\hspace{0.17em}\times \hspace{0.17em}\mathrm{T}1\mathrm{W}\_\mathrm{Skewness}\hspace{0.17em}+\hspace{0.17em}1.10\hspace{0.17em}\times \hspace{0.17em}\mathrm{T}2\mathrm{W}\_\mathrm{GLDM}\_\mathrm{SDLGLE}$$

### External validation of the prediction model

**Fig. 4:**
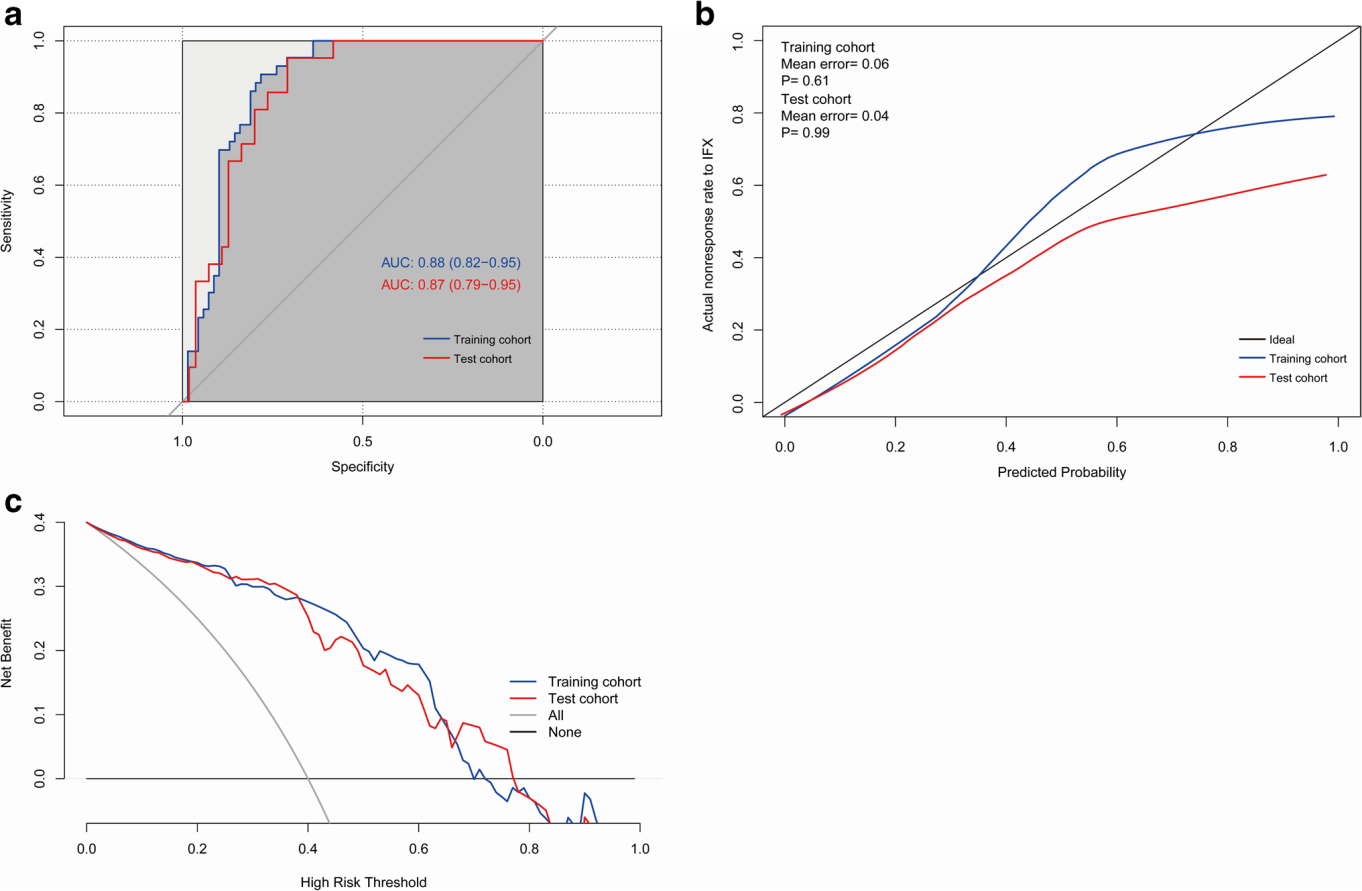
Model performance in the training and validation cohorts. **a** Receiver operating curve. **b** Calibration plots. **c** Decision curve analyses

The AUCs of models 1 and 2 were 0.87 (95% confidence interval (CI): 0.79–0.95) and 0.83 (95% CI: 0.74–0.92), respectively, and the results of DeLong’s test showed that the AUCs of the two models were similar (*p* = 0.06). Furthermore, the addition of BMI and disease phenotype in the model did not improve discriminatory power through IDI analysis (IDI: − 0.04, *p* = 0.05). Considering the model efficiency and clinical applications, the radiomic model was proposed as the optimal prediction model. To further validate the model performance, we evaluated model discrimination, calibration, and clinical effectiveness in both the training and validation cohorts. ROC analysis is presented in Fig. 4A; the AUC of model in the training cohort was 0.88 (95% CI: 0.82–0.95). DeLong’s test simultaneously identified that the classification ability of the radiomic model was significantly superior to that of DWI_GLSZM_SALGLE (training cohort: *p* < 0.000; validation cohort: *p* = 0.003), T1W_Firstorder_Skewness (training cohort and validation cohort: *p* < 0.000), and T2W_GLDM_SDLGLE (training cohort: *p* = 0.001; validation cohort: *p* = 0.012). The mean error (training: 0.06, test: 0.04) in calibration curves and the *p* value of the Hosmer–Lemeshow test (training cohort: 0.61, validation cohort: 0.99) demonstrated that the predictive probability of IFX efficacy was consistent with its actual probability (Fig. 4B). The results of DCA revealed that the model brought clinical benefit with reasonable threshold probabilities ranging from 2 to 70% in the training cohort and 2 to 78% in the validation cohort (Fig. 4C).

### Radiomic model performance in different disease locations, phenotypes, severity, MRE scanners, and hospital campuses

#### Different disease locations

The patients were classified into small intestine and colonic CD. The performance of the radiomic model in different disease locations is presented in Table 4. The AUCs achieved for small intestine and colonic CD were 0.90 and 0.87, respectively, in the training cohort and 0.80 and 0.92 in the validation cohort, respectively. DeLong’s test showed that this model achieved a favorable diagnostic ability in identifying IFX nonresponder patients with CD (training cohort: *p* = 0.61; validation cohort: *p* = 0.18). The accuracies of the model were > 0.69 among different CD locations.

**Table 4:** Radiomic model performance in different disease locations, phenotypes, severity, MRE scanners, and hospital campuses

	AUC	Accuracy	SE	SP	PPV	NPV
Training cohort						
Small intestine (52)	0.9	0.81	0.77	0.83	0.77	0.83
Colon (60)	0.87	0.80	0.81	0.79	0.68	0.89
Test cohort (76)						
Small intestine (35)	0.80	0.69	0.62	0.72	0.57	0.76
Colon (41)	0.92	0.88	0.75	0.91	0.67	0.94

#### Different disease phenotypes

No significant differences in the AUCs of the radiomic model between inflammatory and stricturing and/or penetrating CD in both the training and validation cohorts (*p* = 0.41 and 0.10, respectively) (Table 5). The model also achieved high accuracy in different phenotypes (training cohort: 0.75 in inflammatory CD and 0.85 in stricturing and/or penetrating CD; validation cohort: 0.84 in inflammatory CD and 0.72 in stricturing and/or penetrating CD).

**Table 5:** Radiomic model performance in different disease locations, phenotypes, severity, MRE scanners, and hospital campuses

	AUC	Accuracy	SE	SP	PPV	NPV
Training cohort						
Inflammatory (57)	0.84	0.75	0.63	0.80	0.56	0.85
Stricturing and/or penetrating (55)	0.90	0.85	0.89	0.82	0.83	0.88
Test cohort						
Inflammatory (44)	0.93	0.84	0.64	0.91	0.70	0.88
Stricturing and/or penetrating (32)	0.78	0.72	0.70	0.72	0.54	0.84

#### Different disease severity

The disease severity of CD was divided into mild and moderate-severe CD based on the HBI score, with a threshold of 7 [21]. ROC analysis revealed no significant differences in the discriminative ability of the radiomic model among different CD severity (all AUCs > 0.80, *p* = 0.86 and 0.49 in training and validation cohorts, respectively). The model also approached adequate accuracy for predicting the effectiveness of IFX therapy in mild and moderate-severe CD (all accuracies > 0.75) (Table 6).

**Table 6:** Radiomic model performance in different disease locations, phenotypes, severity, MRE scanners, and hospital campuses

	AUC	Accuracy	SE	SP	PPV	NPV
Training cohort						
Mild (71)	0.87	0.76	0.65	0.81	0.63	0.83
Moderate to severe (41)	0.86	0.88	0.95	0.81	0.83	0.94
Test cohort						
Mild (58)	0.88	0.79	0.63	0.86	0.63	0.86
Moderate to severe (18)	0.80	0.78	0.80	0.77	0.57	0.91

#### Different MRE scanners

We found there was no significant differences in the AUCs of the model between the two MRE scanners (all AUCs > 0.80, all *p* > 0.80 in the training and validation cohorts). The predictive accuracy of the model was verified among different MRE scanners and cohorts (accuracy > 0.75) (Table 7).

**Table 7:** Radiomic model performance in different disease locations, phenotypes, severity, MRE scanners, and hospital campuses

	AUC	Accuracy	SE	SP	PPV	NPV
Training cohort						
3.0-T Philips’s scanner (78)	0.88	0.79	0.73	0.83	0.73	0.83
1.5-T GE scanner (34)	0.89	0.82	0.92	0.76	0.71	0.94
Test cohort						
3.0-T Philips’s scanner (52)	0.87	0.79	0.69	0.83	0.65	0.86
1.5-T GE scanner (24)	0.87	0.79	0.60	0.84	0.50	0.89

#### Different hospital campuses

All the AUCs of the model were > 0.75, and *p* > 0.1 among different hospital campuses. Additionally, we confirmed that the model had an accuracy > 0.69 in patients from east and west campuses (Table 8).

**Table 8:** Radiomic model performance in different disease locations, phenotypes, severity, MRE scanners, and hospital campuses

	AUC	Accuracy	SE	SP	PPV	NPV
Training cohort						
East campus (70)	0.86	0.77	0.72	0.80	0.72	0.80
West campus (42)	0.92	0.86	0.93	0.82	0.77	0.96
Test cohort						
East campus (50)	0.92	0.84	0.67	0.91	0.77	0.86
West campus (26)	0.78	0.69	0.67	0.70	0.40	0.87

### Effectiveness of IFX with time between different risk groups

**Fig. 5:**
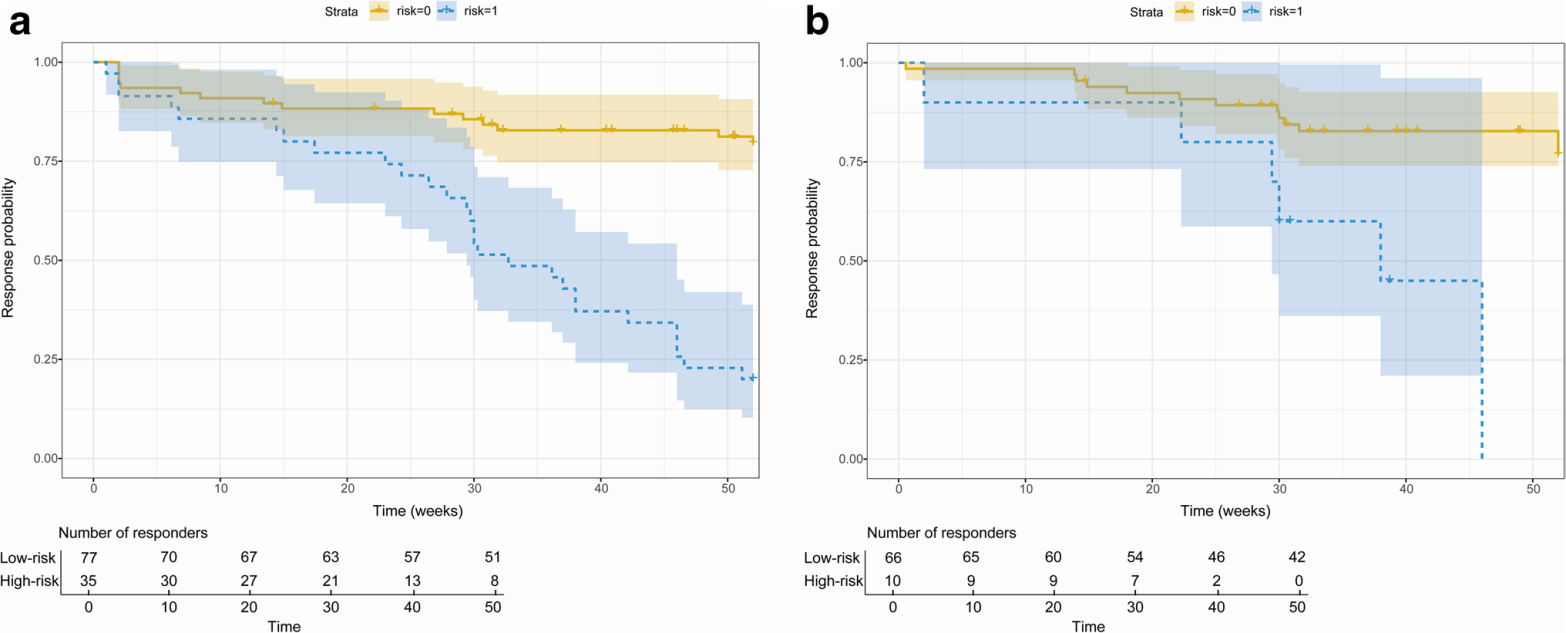
Kaplan–Meier curves of the IFX efficacy between the low- and high-risk groups defined by radiomic model in the training (**a**) and validation (**b**) cohorts

Patients were identified as low- and high-risk groups for IFX nonresponse, with an optimal cutoff of 0.39. Therefore, 35 (31.25%) and 10 (13.16%) patients were classified into the high-risk group from the training and validation cohorts, respectively. Kaplan–Meier analysis and log-rank test showed that high-risk patients were strongly associated with a higher incidence of IFX nonresponse as well as shorter effective time of IFX in both the training (*p* < 0.0001) and validation (*p* = 0.001) cohorts (Fig. 5). In the training cohort, 50% of high-risk patients showed nonresponse to IFX at 33 weeks after therapy, which was 38 weeks in the validation cohort.

## Discussion

Consistent with previous studies, 27.6–38.4% of patients with CD in our study developed either PLR or SLR to IFX therapy [1]. Our study investigated the predictive role of MRE features in CD treatment and established a radiomic model that could assess the probability of IFX therapy failure. Patients were divided into low- and high-risk groups by risk stratification; the nonresponse rate differed significantly between the two groups within 52 weeks after IFX treatment.

As a radiation-free measurement, MRE has become the preferred technique for follow-up of CD according to the ECCO guidelines [22]. Jordi et al found that the absence of creeping fat and severe inflammatory lesions at baseline were negative predictors of radiological healing with anti-TNF therapy in CD, and the model approached predictive capacity with an AUC of 0.78 [11]. In stricturing CD, patients with low ADC values from DWI were more likely to develop anti-TNF failure within 12 months (AUC = 0.81) [12]. Unlike MRI findings such as bowel wall thickness and ulcerations, TA is a new approach to quantify the gray scale, voxel, and position of the images, and reflects the heterogeneity and histopathological variation of lesions [23]. The promising roles of MRI-TA in diagnosis and therapy assessment of cancers have been investigated [24–26]. While few studies have investigated the use of TA in CD, only one study proposed that MRE features in T1WI could accurately differentiate strictures and fibrosis in CD (AUC = 0.995) [15]. Our previous study developed a model based on computed tomography enterography (CTE) texture features for the identification of SLR to IFX in CD [27]. This study aimed to explore the MRE-TA features as predictors of IFX treatment outcomes in patients with CD.

We obtained the candidate features from post-T1WI, T2WI, and DWI sequences, which are mandatory for CD monitoring as recommended by ECCO [8], and the selected texture features were DWI_GLSZM_SALGLE, T1W_Skewness, and T2W_GLDM_SDLGLE. Studies have found the value of GLSZM was associated with the prognosis in tumor [28, 29]. The negative value of skewness indicates the lower brightness of the histogram, and the greater absolute value indicates the higher degree of deviation [19]. The increased value of T2W_SDLGLE may be associated with a higher risk of malignant tumors and disease recurrence [20]. In our study, the loss of response to IFX therapy exhibited an elevated T2_GLDM_SKDLGLE value and decreased levels of DWI_GLSZM_ALSGLE and T1W_Skewness. The T1W_Skewness value was negative, with a higher absolute value compared to that of responders. These differences suggest that lesions in nonresponders show increased heterogeneity and intestinal fibrosis.

The AUC and IDI analysis revealed that the radiomic model had greater discriminative ability compared to the combined model. The radiomic model also showed superior accuracy and clinical utility in external validation. Of note, the robustness of this radiomic model for identifying the risk of IFX nonresponse was not significantly affected by the location, phenotype, disease activity of CD, and various hospital campuses and MRE scanners. Hence, our radiomic model exhibits excellent stability and generalization. To increase the clinical applicability of this model, patients were allocated into low- and high-risk groups for IFX nonresponse with a radiomic score of 0.39; the high-risk patients were observed to have a significantly higher rate and shorter mean duration of IFX nonresponse within 52 weeks.

Our model performed well in the early identification of IFX nonresponse; however, this study has some limitations. First, the limited sample size was a drawback. 134 patients out of a total of 332 CD patients were not enrolled in our study for various reasons, including 63 patients who had anti-TNF exposure, 59 patients who were unable to receive 5 mg/kg IFX therapy, and other 22 patients due to insufficient clinical and imaging data. The second limitation was a retrospective nature, especially the evaluation of the models’ effectiveness in the test cohort. Although the sample size in our study was the largest ever in a radiomic TA of CD therapy, a larger population is needed to strengthen our model in a further prospective study. Third, MRE is a time-consuming and expensive technique, having high requirements on the operator technology and patient status, and patients with acute symptoms should undergo CT. Another limitation is that the features of T2FS sequence were not included in the radiomic model. It is acknowledged that T2W-FS sequence could play an important role to identify acute inflammation; however, the portion of fibrosis or fat was barely indistinguishable on T2W-FS sequence because it eliminated their signal difference, and the results of LASSO regression showed the features of T2FS sequence contributed little to predicting the loss of response. Furthermore, manual VOI segmentation is time consuming and inaccurate. We propose the development of automatic approaches to select VOIs based on deep learning. Finally, a more accurate and stable model comprising various genetics, clinicopathologic characteristics, and molecular biomarkers should be investigated in the future.

## Conclusion

Our study established a radiomic model as an effective approach for estimating the risk of nonresponse to IFX in CD. This model successfully divided patients into low- and high-risk groups for IFX therapy failure and may be conveniently used to guide IFX therapy and individualized treatment strategies for patients with CD.

## Supplementary Information

Below is the link to the electronic supplementary material.

Supplementary file1 (PDF 181 KB)

## Abbreviations

ADC: Apparent diffusion coefficient
AIC: Akaike information criterion
AUC: Area under the curve
BMI: Body mass index
CD: Crohn’s disease
CV: Cross-validation
DCA: Decision curve analysis
DWI: Diffusion-weighted imaging
ECCO: European Crohn’s and Colitis Organization
HBI: Harvey-Bradshaw Index
ICC: Intraclass correlation coefficient
IDI: Integrated discrimination improvement
IFX: Infliximab
LASSO: Least absolute shrinkage and selection operator
MRE: Magnetic resonance enterography
MRI: Magnetic resonance imaging
PLR: Primary loss of response
ROC: Receiver operating characteristic
SES-CD: Simple endoscopic score for CD
SLR: Secondary loss of response
T1WI: T1-weighted fat-suppressed images
T2WI: T2-weighted image
T2WI-FS: T2-Weighted image with fat suppression
TA: Texture analysis
TNF: Tumor necrosis factor
VOI: Volume-of-interest

## Declarations

### Guarantor

The scientific guarantor of this publication is Jun Shen.

### Statistics and biometry

No complex statistical methods were necessary for this paper.

### Methodology

• retrospective

• diagnostic or prognostic study

• multicenter study
