# Supplementary material for: Infliximab response associates with radiologic findings in bio-naïve Crohn’s disease
Source: Eur Radiol. 2023 Mar 16;33(8):5247–57. doi: 10.1007/s00330-023-09542-y (PMC10326128; doi:10.1007/s00330-023-09542-y)
Supplement: Supplementary file 1 — Supplementary file1 (PDF 181 KB) [file 330_2023_9542_MOESM1_ESM.pdf]

ELECTRONIC SUPPLEMENTARY MATERIAL

Infliximab Response Associates with Radiologic Findings in Bio-naïve Crohn's Disease

Supplementary Table 1.

(a) Parameters of Pulse Sequences for MR Enterography (1.5T)

Parameter	FIESTA Coronal	T2WI SSFSE Coronal	T2WI-FS SSFSE Axial	T1WI Dual Echo Axial	T1 LAVA-Flex BH contrast Coronal	DWI (b=600) Axial
Repetition time/echo time (msec)	3.5/1.54	1500/160	435/70	180/2.1(3.4)	6.7/2	(6208/min)
Flip angle (degrees)	80	—	90	80	12	90
Matrix	128×224	224×256	172×194	170×256	192×320	128×130
Nex	1	1	1	1	1	1
Slice thickness (mm)	5	6	6	6	3	6
spacing(mm)	1	1	0.6	0.6	0	0.6

(b) Parameters of Pulse Sequences for MR Enterography (3T)

Parameter	BTFE Coronal	T2WI TSE Coronal	T2WI-FS TSE Axial	T1WI TFE IP Axial	T1 mDIXON contrast Coronal	DWI (b=1000) Coronal
Repetition time/echo time (msec)	3.5/1.48	875/100	shortest/70	10/2.3	3.8/1.35	898/64
Flip angle (degrees)	90	90	90	15	10	90
Matrix	364x299	332×239	240×355	268×176	308x272	132x134
Nex	1	2	1	1	1	3
Slice thickness (mm)	6	5	6	5	2	5
spacing(mm)	0	0	0.6	0.5	-2	0.5

Supplementary Table 2. Features included in MR enterography.

Sequence	Matrix	Features
Contrast-enhanced T1-weighted-imaging (T1WI)	Shape	Shape_Elongation
		Shape_Maximum2DDiameterColumn
		Shape_Maximum2DDiameterRow
		Shape_Maximum2DDiameterSlice
		Shape_MeshVolume
		Shape_MinorAxisLength
		Shape_Sphericity
		Shape_SurfaceVolumeRatio
	First-order	Firstorder_Kurtosis
		Firstorder_Minimum
		Firstorder_Skewness
		Firstorder_TotalEnergy
	Grey level co-occurrence matrix (GLCM)	GLCM_ClusterShade
		GLCM_Correlation
		GLCM_Idmn
		GLCM_MCC
	Gray level difference matrix (GLDM)	GLDM_DependenceNonUniformity
		GLDM_LargeDependenceLowGrayLevelEmphasis
		GLDM_LowGrayLevelEmphasis
		GLDM_SmallDependenceLowGrayLevelEmphasis
	Gray level run length matrix (GLRLM)	GLRLM_GrayLevelNonUniformity
		GLRLM_GrayLevelNonUniformityNormalized
	Gray level size zone matrix (GLSZM)	GLSZM_LargeAreaHighGrayLevelEmphasis
		GLSZM_SmallAreaEmphasis
		GLSZM_ZoneVariance
	Neighborhood gray-tone difference matrix (NGTDM)	NGTDM_Busyness
		NGTDM_Coarseness
		NGTDM_Strength
		NGTDM_Contrast
Contrast-enhanced T2-weighted-imaging (T2WI)	Shape	Shape_Elongation
		Shape_MinorAxisLength
		Shape_Sphericity
		Shape_SurfaceVolumeRatio
		Shape_VoxelVolume
	First-order	Firstorder_Energy
		Firstorder_Kurtosis
		Firstorder_Minimum
		Firstorder_Skewness
	Grey level co-occurrence matrix (GLCM)	GLCM_ClusterShade
		GLCM_Correlation
		GLCM_Idmn

		GLCM_Imc2
		GLCM_MCC
	Gray level difference matrix (GLDM)	GLDM_LargeDependenceHighGrayLevelEmphasis
		GLDM_SmallDependenceLowGrayLevelEmphasis
	Gray level run length matrix (GLRLM)	GLRLM_RunVariance
		GLRLM_ShortRunHighGrayLevelEmphasis
	Gray level size zone matrix (GLSZM)	GLSZM_GrayLevelNonUniformity
		GLSZM_LargeAreaLowGrayLevelEmphasis
		GLSZM_SmallAreaEmphasis
	Neighborhood gray-tone difference matrix (NGTDM)	NGTDM_Coarseness
		NGTDM_Complexity
		NGTDM_Contrast
Diffusion-weighted-imaging (DWI)	Shape	Shape_Elongation
		Shape_Maximum2DDiameterRow
		Shape_Maximum2DDiameterSlice
		Shape_MinorAxisLength
		Shape_SurfaceVolumeRatio
	First-order	Firstorder_Kurtosis
		Firstorder_Minimum
		Firstorder_Skewness
	Grey level co-occurrence matrix (GLCM)	GLCM_Idmn
		GLCM_Imc1
		GLCM_InverseVariance
		GLCM_Correlation
	Gray level difference matrix (GLDM)	GLDM_DependenceNonUniformityNormalized
		GLDM_DependenceVariance
		GLDM_SmallDependenceLowGrayLevelEmphasis
	Gray level run length matrix (GLRLM)	GLRLM_GrayLevelNonUniformity
		GLRLM_RunEntropy
		GLRLM_RunLengthNonUniformityNormalized
		GLRLM_ShortRunLowGrayLevelEmphasis
	Gray level size zone matrix (GLSZM)	GLSZM_GrayLevelNonUniformity
		GLSZM_LargeAreaHighGrayLevelEmphasis
		GLSZM_LargeAreaLowGrayLevelEmphasis
		GLSZM_SizeZoneNonUniformity
		GLSZM_SizeZoneNonUniformityNormalized
		GLSZM_SmallAreaEmphasis
		GLSZM_SmallAreaLowGrayLevelEmphasis
	Neighborhood gray-tone difference matrix (NGTDM)	NGTDM_Busyness
		NGTDM_Coarseness
		NGTDM_Complexity

T2-Weighted Sequence with Fat Suppression (T2WI-FS)	Shape	shape_Elongation
		shape_MajorAxisLength
		shape_Maximum2DDiameterColumn
		shape_Maximum2DDiameterRow
		shape_Sphericity
	First-order	firstorder_Energy
		firstorder_Kurtosis
		firstorder_RobustMeanAbsoluteDeviation
		firstorder_Skewness
	Grey level co-occurrence matrix (GLCM)	GLCM_ClusterShade
		GLCM_Correlation
		GLCM_DifferenceVariance
		GLCM_Idm
		GLCM_Idmn
		GLCM_Imc2
		GLCM_InverseVariance
		GLCM_MCC
	Gray level run length matrix (GLRLM)	GLRLM_GrayLevelNonUniformityNormalized
		GLRLM _RunEntropy
		GLRLM _RunVariance
	Gray level size zone matrix (GLSZM)	GLSZM _LargeAreaHighGrayLevelEmphasis
		GLSZM _LargeAreaLowGrayLevelEmphasis
		GLSZM _SizeZoneNonUniformity
		GLSZM _SmallAreaEmphasis
		GLSZM _SmallAreaLowGrayLevelEmphasis
	Neighborhood gray-tone difference matrix (NGTDM)	NGTDM _Busyness
		NGTDM _Coarseness
		NGTDM _Contrast
		NGTDM _Strength

Supplementary Table 3. Univariate regression analysis of clinical variables in the training cohort

Characteristics	Response (n=69)	Loss of response (n=43)	P.value
Sex (male/female, n)	49/20	31/12	0.902
Age, years, (mean±SD)	30.81±11.25	32.16±10.89	0.529
Duration of the disease, months, median (IQR)	33.14 (4-42)	40.28 (6-60)	0.441
BMI, kg/m^2, (mean±SD)	20.50±3.19	19.14±2.64	0.024*
Prior surgery, n (%)	16 (23.19%)	15 (34.88%)	0.181
Smoking at first dose of IFX, n (%)	4 (5.80%)	2 (4.65%)	0.794
Location of Specimen, n (%)			0.428
Small bowel	30 (43.48%)	22 (51.16%)	
Colon	39 (56.52%)	21 (48.84%)	
Phenotype, n (%)			0.024*
Inflammatory	41 (59.42%)	16 (37.21%)	
Stricturing and/or penetrating	28 (40.58%)	27 (62.79%)	
Perianal fistulas, n (%)	47 (68.12%)	27 (62.79%)	0.563
CRP, mg/L, median (IQR)	13.79 (0.55-15.1)	10.63 (0.25-16.7)	0.445
ESR, mm/h, median (IQR)	21.32 (6-30)	25.72 (6-29)	0.532
Hb, g/L, median (IQR)	127.52 (109.5-138.5)	125.11 (112-136)	0.712
Alb, g/L, median (IQR)	40.20 (37-43.7)	41.17 (37.5-46.4)	0.431
PLT, $\times 10^9$/L, median (IQR)	298.8 (220.5-352)	265.37 (197-322)	0.082
HBI, median (IQR)	6.3 (4-8)	6.98 (5-8)	0.196
SES-CD, median (IQR)	11.19 (6-17)	12.72 (8-17)	0.101

*, P value<0.05; BMI, Body Mass Index; Alb, Albumin; CRP, C-reactive protein; ESR, Erythrocyte Sedimentation Rate; Hb, Hemoglobin; PLT, Platelets; HBI, Harvey Bradshaw indices; SES-CD, simple endoscopic score for CD; sd, standard deviation; IQR, interquartile range.
